# Development of Palpable Purpura in a Patient With Infective Endocarditis: A Case Report

**DOI:** 10.7759/cureus.63601

**Published:** 2024-07-01

**Authors:** Jordan Lane, John M Read, Zalmi Rahmany, Kelsey Reely, Courtney M Hicks, David E Martin

**Affiliations:** 1 Internal Medicine, Unity Health, Searcy, USA; 2 Infectious Disease, Unity Health, Searcy, USA; 3 Physiology, Unity Health, Searcy, USA

**Keywords:** intravenous drug use, staphylococcus aureus, vasculitis, purpura, infective endocarditis

## Abstract

Infective endocarditis (IE) can present with a variety of signs and symptoms, including skin lesions. The few papers describing a relationship between IE and vasculitis are split between IE being able to mimic vasculitis and between IE indeed being associated with a vasculitis involving the skin, kidney, gastrointestinal tract, or peripheral nerves. It is important for clinicians to distinguish between an isolated vasculitis, infective endocarditis, and IE-associated vasculitis because the treatments and outcomes are different. We report a case of a patient with a history of intravenous (IV) drug use who initially presented with chest pain, was started on vancomycin following diagnosis of methicillin-resistant *Staphylococcus aureus* (MRSA) IE, left against medical advice (AMA), and then returned to the hospital due to development of a purpuric rash. We contend that while he did not have a skin biopsy due to time delay, his symmetrically distributed purpura was consistent with cutaneous vasculitis. His symptoms, including his rash and an acute kidney injury (AKI), improved with antibiotics to treat the endocarditis.

## Introduction

Infective endocarditis (IE) has been associated with the transient development of autoimmune markers, vasculitis, and glomerulonephritis, suggesting an overactivation of the immune system in response to bacteria [1-14]. In a 2020 review of *Staphylococcus aureus* IE associated with vasculitis or glomerulonephritis, the clinical signs and symptoms that raised suspicion for an autoimmune process were purpura or an acute kidney injury (AKI) [1]. What can complicate the significance of rashes in IE is that besides the quintessential Osler nodes, Janeway lesions, and Roth’s spots of endocarditis, rashes can also occur from septic emboli, IE-associated vasculitis, and from the antibiotics used to treat IE. A few cases have been reported of patients developing small-vessel vasculitis after starting vancomycin [15]. Purpura is significant in patients with IE, as biopsies have demonstrated septic embolic or leukocytoclastic vasculitis [4]. As for the treatment of IE or IE-associated vasculitis, antibiotics are generally sufficient; however, there are instances where immunosuppressive therapy can be beneficial or harmful. Here, we provide a brief literature review of the diagnosis and management of IE and IE-associated vasculitis. We also report a case of a 40-year-old man with a history of intravenous (IV) drug use who was diagnosed with definite IE from methicillin-resistant *S. aureus* (MRSA), left against medical advice (AMA) after two days of vancomycin, and returned to the hospital after developing purpura; he was found to have an AKI on re-admission.

## Case presentation

**Figure 1:**
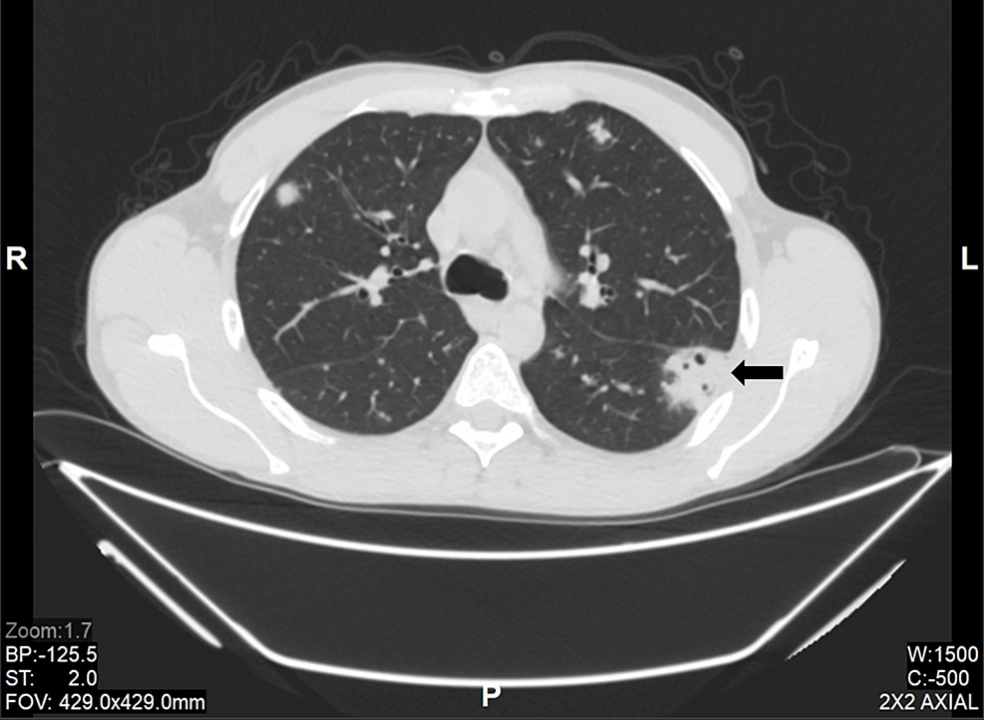
Non-contrast CT of the thorax, lung window Pulmonary nodules with fluffy margins are located in the periphery, and a cavitating lesion is also seen (arrow).

A 40-year-old Caucasian man with a history of IV methamphetamine drug use presented to the Emergency Department (ED) complaining of fever. Blood cultures were drawn, and he left AMA before being seen by a physician. He then returned to the ED four days later, complaining of persistent fever, malaise, and diffuse chest pain. The previously collected blood cultures had grown MRSA. Of note, the patient stated that his last IV drug use was one month before this. Laboratory values were significant for a white blood cell (WBC) count of 19.7 k/µL and an elevated absolute neutrophil count of 16.06 k/µL. Troponin levels were negative, and an electrocardiogram did not show significant change from prior measurements. A non-contrast computed tomography (CT) of the thorax showed fluffy nodularities suspicious for septic emboli (Figure 1). The patient was then started on antibiotics with a weight-based dose of vancomycin IV infusion and admitted to the hospital. A transthoracic echocardiogram (TTE) was performed. Although no vegetations were found on the echocardiogram, the patient was presumed to have definite endocarditis, as defined by having one major (blood culture typical for IE) plus three minor criteria (fever, vascular phenomena of septic pulmonary emboli, and predisposition with IV drug use) under the Modified Duke Criteria for infective endocarditis [16]. Since the patient’s hospitalization, the Modified Duke Criteria have been updated to the Duke-International Society for Cardiovascular Infectious Diseases (Duke-ISCVID) Criteria [17].

A transesophageal echocardiogram (TEE) was planned for the following day. However, the patient again left against medical advice. At the time the patient left, four doses of vancomycin had been given with some improvement in the patient’s WBC count (11.6 k/µL).

**Figure 2:**
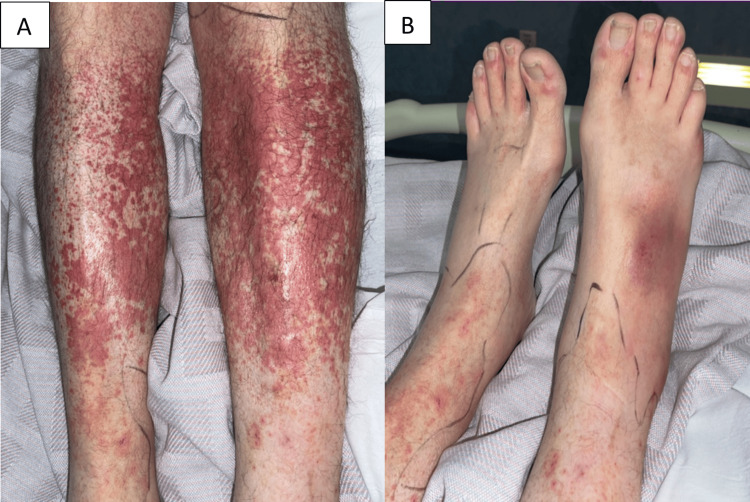
Rashes from Day 2 of patient's second hospitalization (A) Purpuric and petechial lesions on the patient’s lower extremities. A marker was used to delineate surrounding erythema. (B) Purpura on his feet.

Four days later, the patient presented back to the ED again with new complaints of edema, pain in his lower extremities, and a rash along the upper and predominately lower extremities, which began the day after leaving AMA. The patient was tachycardic with a pulse of 116 bpm but afebrile on the second presentation. On physical examination, there was a palpable purpuric and petechial rash along the bilateral distal lower extremities (Figures 2A, 2B) and increased edema in bilateral hands.

At that time, the WBC count was within the normal range at 9.3 k/µL, although ESR and CRP were elevated at 73 mm/h and 4.2 mg/dL, respectively. The platelet count was elevated at 503 k/µL. Aspartate and alanine aminotransferase levels were within normal limits at 22 U/L and 21 U/L, respectively. Coagulation studies were also normal with prothrombin time of 11.3 s, international normalized ratio of 1.0, and partial thromboplastin time of 29.5 s. Urinalysis was significant for 2+ protein, a WBC count of 42/hpf, and trace hematuria with an RBC count of 10/hpf. Spot urine protein was 195.9 mg/dL and spot urine creatinine was 494.8 mg/dL, giving an elevated urine protein to creatinine ratio of 0.4. His creatinine was elevated at 2.4 mg/dL, from baseline 1.0 mg/dL on previous admission, and HIV screening was negative. Due to concerns about small-vessel vasculitis associated with the recent vancomycin use, linezolid IV infusion was initiated in the ED for recently diagnosed MRSA endocarditis. The patient was then readmitted to the inpatient unit for further treatment. Within 12 hours, his kidney function had improved, with decreased creatinine, from 2.4 to 1.5 mg/dL.

The following day, linezolid was discontinued, and vancomycin was restarted, considering the new symptoms began following vancomycin discontinuation. Values of complement C3 and C4 were found to be below the normal range, at 68 mg/dL and 15 mg/dL, respectively. The Cardiology department was consulted and it was decided that a transesophageal echocardiogram was not needed since it would not change the management of his definite endocarditis. After a consultation with the Infectious Disease department, antibiotic therapy with vancomycin was continued, and over the next 10 days, the new onset purpura and petechiae improved. Creatinine levels returned to the baseline level of 1.0 mg/dL, indicating the resolution of AKI. The patient was subsequently discharged with an outpatient Infectious Disease follow-up appointment, as well as prescriptions for sulfamethoxazole-trimethoprim 1600-320 mg, orally twice a day, and doxycycline 100 mg, orally twice a day, to complete a six-week course of antibiotics from the first negative blood cultures obtained in the setting of infective endocarditis.

## Discussion

IE is an infection of endocardial tissue, especially of the cardiac valves, and it should be suspected in a patient with signs and symptoms of fever, shortness of breath, Osler nodes, Janeway lesions, Roth’s spots, new murmurs, or persistent bacteremia [18]. TTE or TEE should be obtained prudently in patients suspected to have IE, with a 60% and 90% respective sensitivity in visualizing vegetations in the heart [19]. Aside from echocardiograms, new imaging like ^18^F-fluorodeoxyglucose positron emission tomography, computed tomography (^18^F-FDG PET-CT), and electrocardiogram-gated cardiac CT angiography are emerging as valuable tools in diagnosing endocarditis, especially in patients with prosthetic valves and implanted cardiac electronic devices [19]. These newer imaging modalities are being incorporated into the latest diagnostic criteria for infective endocarditis. The ISCVID has recently updated the Duke Criteria to the Duke-ISCVID Criteria, which allow for determining definite, possible, and rejected infective endocarditis based on clinical and pathologic criteria [17]. These updated criteria have several changes from the 2000 Modified Duke Criteria, including adding categories to the major criteria and changing the auscultation of new valvular regurgitations from a major criterion to a minor one [16]. The major microbiologic criteria have been expanded to include a broader list of typical pathogens and allow for PCR or immunofluorescence evidence of certain organisms to qualify instead of solely relying on blood cultures [17]. The major imaging criteria have been updated from just echocardiography to now incorporate cardiac CT and ^18^F-FDG PET-CT in identifying IE. As for the diagnosis of definite IE, the clinical criteria remain two major criteria, one major criterion plus three minor criteria, or five minor criteria. Diagnosing possible IE remains as one major criterion plus one minor criterion or three minor criteria.

Our patient had definite IE based on one major clinical criterion (positive blood culture with a microorganism that commonly causes IE) and three minor clinical criteria (fever, predisposition with IV drug use, and vascular phenomena with radiological evidence of septic pulmonary emboli). The Cardiology and Infectious Disease services consulted also agreed with the diagnosis of definite IE. His purpura on re-admission was worrisome, which led to a discussion of the rash being a sequela of IE, a vasculitis, or adverse drug reaction from vancomycin during the first hospitalization.

It is important to be familiar with the skin findings mentioned above for IE since rashes are evident to the patient and care team, and they can clue in practitioners to IE. The prevalence of skin findings in IE varies, especially with relatively higher rates described in papers from the 20th century [20-23]. Still, an observational and prospective study from 2014 found that 8.0% of patients had purpura, 2.7% had painful Osler nodes, and 1.6% had painless Janeway lesions [24]. More severe rashes can also be seen in IE, such as the hematologic emergency of purpura fulminans, where blood clots in small vessels lead to skin necrosis [25]. The general purpura in IE typically occurs on patients’ backs, legs, mucosa, or near the clavicles. Biopsies of the purpura have revealed the cause to be septic embolic or leukocytoclastic vasculitis, an inflammatory disease process where neutrophils and their debris are seen under microscopy to infiltrate the walls of small blood vessels [4]. Leukocytoclastic vasculitis is also known by other terms, such as hypersensitivity vasculitis and cutaneous small-vessel vasculitis [26]. *S. aureus* is the main culprit of IE; the first described account of vasculitis from *S. aureus* infective endocarditis is from 1976 [2,27,28]. When the bacterium causes vasculitis, the organs most commonly affected are the kidneys, skin, gastrointestinal tract, and peripheral nerves. Kidney involvement has been described as AKI with hematuria and/or proteinuria, membranoproliferative glomerulonephritis, crescentic glomerulonephritis, and pauci-immune glomerulonephritis [1,9]. Since 1976, biopsies of patients with staphylococcal endocarditis and concurrent purpura have also shown leukocytoclastic vasculitis [1,2,5]. Some of these skin and kidney biopsies also demonstrated deposition of immunoglobulin A and complement C3, with some authors arguing for, and some against, the diagnosis of Henoch-Schönlein purpura, which clinically presents with purpura, abdominal pain, and lower extremity arthralgias [3,8]. Hence, purpura in patients with infective endocarditis should raise concern for small-vessel vasculitis.

The pathophysiology of IE and associated leukocytoclastic vasculitis is not fully known, but several autoantibodies have been detected in patients with IE. It is thought that B-cell exposure to bacteria may lead to the subsequent production of autoantibodies such as an antinuclear antibody, antineutrophil cytoplasmic antibody (ANCA), rheumatoid factor, and anti-β-2-glycoprotein I, followed by the activation of neutrophils and downstream inflammation [9]. ANCA is the most reported autoantibody in the setting of IE, with 8%-33% positivity in patients with IE, and the bacteria that mainly induce ANCA production are *Staphylococcus* and *Streptococcus* species, but *Mycobacterium tuberculosis*, *Legionella*, and *Bartonella* are also known to cause ANCA production [9,10]. At an early stage, leukocytoclastic vasculitis and other secondary autoimmune processes can resolve with only antibiotics to treat the IE. If cardiac valves are damaged, surgery may also be needed along with antibiotics [19]. If the endocarditis is left unchecked, the patient may develop a more systemic vasculitis, as evidenced by development/progression/relapse of skin, gastrointestinal, or renal involvement, at which point the addition of immunosuppressive therapy may be necessary to treat end-organ damage [1,8,10].

While our patient did not have a biopsy, his symmetrically distributed purpura closely resembles that depicted in studies with microscopically diagnosed leukocytoclastic vasculitis in patients with IE, which combined with a previously stated idea that cutaneous vasculitis can be diagnosed clinically [4] led us to conclude that our patient had cutaneous vasculitis in the setting of IE [1,5,11]. Ideally, a skin biopsy of the purpura would have been done, but this was only considered several days into his second hospital admission, and at that point, his rashes were improving and a biopsy was anticipated to be of little value by then. His serum complement levels were also low, which is a finding especially seen in patients with IE and leukocytoclastic vasculitis [6]. His purpura, among other sequelae of his IE, responded well to only antibiotics, likely because his inflammatory activity was still in an early and quickly reversible stage. As his kidney function had rapidly normalized, risks of a kidney biopsy heavily outweighed benefits.

As already mentioned, the treatment of endocarditis with or without vasculitis is antibiotics, with possible valve repair or replacement if the cardiac valves are damaged. Since IE is an infectious process, the addition of immunosuppressive therapy carries the risk of worsening the infection, and it should be considered on a case-by-case basis in patients with endocarditis-associated vasculitis. Immunosuppressants likely only have a net benefit if the patient has worsening systemic signs/symptoms such as of the kidneys or gastrointestinal tract or if their condition relapses despite treatment with antibiotics; this suggests a glomerulonephritis or systemic vasculitis that may have life-threatening effects if not managed with immunosuppression, which typically involves corticosteroids, but may also include IV immunoglobulin or cyclophosphamide [1,9,10].

The antibiotic regimen for IE generally entails empiric coverage of *S. aureus*, *Streptococcus* species, and *Enterococcus* species. Guidelines suggest using empiric IV vancomycin or daptomycin, depending on local resistance patterns, with or without IV ceftriaxone or cefazolin for additional coverage of the *Streptococcus* species [29]. In patients with a prosthetic valve, the empiric therapy is the same unless the valve was placed within three months, in which case coverage for *Pseudomonas* is desired with IV cefepime, piperacillin-tazobactam, or a carbapenem as a second agent to pair with vancomycin or daptomycin. Once the causative bacterium is known, the antibiotics can be tailored accordingly. For most cases of endocarditis, the duration of antibiotics is six weeks. However, the duration can be as short as two weeks, depending on the causative bacterium and if a single antibiotic is used versus a combination of antibiotics. Our patient had first left AMA from our ED after blood cultures were drawn, and these had resulted in MRSA infection by the time of his first hospitalization, which is why IV vancomycin was used. He was discharged from the second hospitalization with oral trimethoprim-sulfamethoxazole and doxycycline for dual coverage of MRSA, as the POET (Partial Oral Treatment of Endocarditis) trial found patients with IE did well transitioning to two oral antibiotics following 10 days of IV antibiotics if the oral agents had different mechanisms of action [30,31]. His IV drug use precluded setting him up with home IV antibiotics through a peripherally placed central catheter (PICC) since he could easily inject through the PICC unless under constant supervision, and it made oral linezolid a suboptimal choice since its combination with methamphetamine could lead to serotonin syndrome. Therefore, after the patient clinically improved and had received 10 days of IV antibiotics, he was medically stable for discharge with oral trimethoprim-sulfamethoxazole and doxycycline for his infective endocarditis, with the expectation that his presumed cutaneous vasculitis would continue to improve as his IE resolved.

## Conclusions

In patients with endocarditis and purpura, clinicians should suspect septic emboli or vasculitis. Our patient with MRSA bacteremia and infective endocarditis-associated purpura was suspected to have a cutaneous vasculitis, and he was successfully treated as such, with just IV antibiotics being the mainstay therapy. Surgery has a role in IE if cardiac valves are damaged. Immunosuppressive therapy can be considered in IE-associated vasculitis if the patient has worsening systemic signs or symptoms, such as of their kidneys or GI tract, or if they relapse despite appropriate antibiotics.
